# Is Multidimensional Poverty Associated to Dementia Risk? The Case of Older Adults in Pakistan

**DOI:** 10.1093/geroni/igae007

**Published:** 2024-02-01

**Authors:** Jean-François Trani, Yiqi Zhu, Soobin Park, Ganesh M Babulal

**Affiliations:** Brown School of Social Work, Washington University in St. Louis, St. Louis, Missouri, USA; National Conservatory of Arts and Crafts, Paris, France; School of Social Work, Adelphi University, Garden City, New York, USA; Brown School of Social Work, Washington University in St. Louis, St. Louis, Missouri, USA; Department of Neurology, Washington University School of Medicine in St. Louis, St. Louis, Missouri, USA

**Keywords:** Alzheimer’s disease, Rural areas, Social and environmental determinants of health, Socioeconomic justice

## Abstract

**Background and Objectives:**

Multidimensional poverty is associated with dementia. We aimed at establishing this association in Pakistan.

**Research Design and Methods:**

A cross-sectional study was conducted in Punjab and Sindh, Pakistan, between March 30, 2002, and August 22, 2022, among adults aged 50 and older. Multidimensional poverty measures were composed of 6 dimensions and 15 indicators. Poverty was compared between adults with and without dementia using the Rowland Universal Dementia Assessment Scale, adjusting for sex, age, marital status, and household size. Associations between dementia and poverty were investigated using a multivariate logistic regression model.

**Results:**

We found that 594 (72.7%), 171 (20.9%), and 52 (6.4%) had no, mild, and moderate-to-severe dementia, respectively. More women than men had dementia (11.4% vs 2.9%). Approximately 40.4% of adults with dementia were found to be deprived in 4 or more dimensions compared to 8.9% without dementia, and the difference in multidimensional poverty between them was 348.6%. Education, health, living conditions, and psychological well-being were the main contributors to poverty. Poverty in 4 or more dimensions was strongly associated with dementia (odds ratio [OR], 5.02; 95% confidence interval [CI], 2.07–12.16) after adjusting for sex, marital status, age, and household size, with greater odds for older women (OR, 2.02; 95% CI, 1.41–2.90).

**Discussion and Implications:**

Our findings suggest that early improvement in social determinants of health through targeted structural policies may prevent dementia later in life. Improving access to free, quality education, health care including mental health care and basic living standards, and employment should reduce the collective risk of dementia.

**Translational Significance:** Social and environmental determinants of health (SEDOH) have been shown to be associated with diseases, but limited research exists investigating how they affect dementia, particularly in low- and middle-income countries. SEDOH in Pakistan are positively associated with dementia in older adults, particularly among women. Planning policies promoting access to quality education, health care including mental health care, better material conditions, and employment may prevent dementia by reducing life-course burden of noncommunicable diseases, both physical and mental. Findings raise social debate about a social justice approach to public policies that could tackle individual poverty by reorganizing existing social arrangements.

## Background and Objectives

Experts predict that the growing aging population at risk of dementia and related cognitive disorders—which is already the seventh leading cause of mortality and a major cause of disability and dependency—will exert profound negative consequences for health care systems, social policy, and in terms of overall economic burden (Nandi et al., 2022; Thrush & Hyder, 2014) for low- and-middle-income countries (LMICs). LMICs are defined by a World Bank classification including countries with a gross national income per capita below $1,135 (low income) between $1,136 and $4,465 (lower middle-income), and $4,466 and $13,845 (upper middle-income). Despite a narrow definition of difference in per capita income (Lencucha & Neupane, 2022), countries in this category do share a higher burden of dementia prevalence that will reach an apogee by 2030 and maintain it for the foreseeable future due to an increase in life expectancy associated with better health care and better coverage of major basic needs (Wu et al., 2016). A recent report by the World Health Organization (WHO) concluded that most countries, specifically LMICs, are not meeting the adopted targets of the global action plan on the public health response to dementia set for 2025 (Cataldi et al., 2022; Lecture Anniversary, 2022; WHO, 2022). The WHO blueprint report emphasized the importance of research on interventions and preventative actions most likely to benefit all persons at risk of dementia, most critically for residents in LMICs, particularly in poorer countries.

### Conceptual Framework

Although research has identified genetic risk factors, our understanding of social and environmental determinants (SEDOH) of dementia is lacking and mechanisms associating these factors to the risk of dementia have hardly been investigated. Currently, modifiable risk factors including absence of education, and social exclusion have been identified (Livingston et al., 2020) where poor SEDOH during the life course such as nutrition, education, health care access, and treatment increase the risk of dementia (WHO, 2022). In the absence of pharmacological treatment for dementia, investigating the pathways between SEDOH and dementia is crucial as potential public and precision health targets for prevention by improving social and environmental contexts through active policies. Timely prioritization of research efforts via SEDOH might lessen the negative impact of the imminent crisis linked to a higher prevalence of dementia and cognitive impairments in LMICs (Parra et al., 2019). In LMICs, adults affected with dementia will represent 60% of the dementia population by 2050 (Patterson, 2018). Families will shoulder the yoke of dementia’s economic burden in the absence of any formal health and social support system (Schatz & Seeley, 2015) threatening an already vulnerable population and socioeconomic system (Casale, 2011; Skovdal et al., 2009). Families will progressively lose contribution of older adults to household supportive tasks like childcare provision. Families will become caregivers of their older members at the cost of other activities, gainful employment for adults, and school attendance for children (Fernqvist, 2015).

### Pakistan, Poverty, and Dementia

Pakistan is a middle-income country with a large population estimated at 240 million, characterized by constant political turmoil, vulnerability to climate crisis, and a growing aging population (United Nations Population Fund, 2023). There is limited knowledge of dementia prevalence. One hospital-based study estimated a rate of prevalence of 3.8% among adults over age 65 (Seetlani et al., 2016). Yet dementia is poorly understood, help-seeking is limited, and older adults with dementia are often stigmatized within families and even communities (Willis et al., 2018).

Pakistan ranks 10th among the most affected countries by climate disasters. In summer 2022, Pakistan witnessed record-breaking climatic variations, with extreme heatwaves followed by monsoon floods that submerged a third of the country, resulting in 1,700 causalities. Over 33 million people were affected, resulting in displacement, massive crop destruction, schools, roads, and other infrastructures damaged or completely lost (Bhutta et al., 2022; Nanditha et al., 2023). Recurring climate disasters affect human development outcomes in a country characterized by 38.3% of the population estimated to be living in multidimensional poverty with a share of deprivation experienced by each poor person averaging 50.9% (United Nations Development Program, 2018). This uniquely vulnerable social and geographical context can lead to a higher risk of dementia.

The capability approach considers that lack of income is insufficient to define poverty, and argues that a multidimensional approach assessing nonmonetary facets of deprivation threatening populations’ well-being in LMICs, particularly basic capabilities such as access to clean water, sanitation, nutrition, education, health care among others, is more relevant (Alkire et al., 2015; Sen, 1999). A growing body of literature shows that poverty is multidimensional (Anand & Sen, 1997; Atkinson, 2003; Oshio & Kan, 2014; Tsui, 2002) and that older adults are particularly at risk of multidimensional poverty in both high (Chan & Chou, 2018; Chen & Leu, 2022; Chou & Lee, 2018; Dhongde, 2017) and LMICs (Mohaqeqi Kamal et al., 2022; Trani et al., 2016). Such a developmental approach centered on deprivation in multiple, proximal dimensions beyond income or consumption is more appropriate to investigate the association between SEDOH and dementia in LMICs (Atkinson, 2003; Sen, 1979). The present study investigates if multiple dimensions of deprivation of older Pakistani adults are associated with a greater incidence of dementia.

## Design and Methods

### Study Design and Sample

A random sample of 1,093 older adults within 750 households from Punjab and Sindh provinces of Pakistan were randomly selected and 834 were consented and interviewed between March 30, 2022 and August 22, 2022. Translation in Urdu and Sindhi of all instruments was conducted by a professional translator from English. Back translations from Urdu and Sindhi to English were conducted by a different professional translator who was blinded to the initial English version. Researchers and translators met to discuss minor discrepancies. The final version was tested and validated for accuracy and both test–retest and inter-rater reliability with a group of 10 older adults in Rahim Yar Khan (Urdu) and Gothki (Sindhi).

### Dementia Outcome

Due to a lack of conventional biomarkers (imaging or cerebrospinal fluid), dementia was screened by the Rowland Universal Dementia Assessment Scale (RUDAS; Storey et al., 2004). We collected information about sociodemographic characteristics, health status, school enrollment, employment, livelihood features, discrimination, depression, stress, and exposure to traumatic events. A 10-day training workshop was conducted to brief staff on study aims and concepts, tools, and interview techniques, and provided mock practice for eight enumerators and two survey supervisors.

### Multidimensional Poverty Measures

Multidimensional poverty is a weighted index composed of six dimensions of well-being, including education, health status, living standards, economic activity, social participation, and psychological health associated with SEDOH (Marmot, 2007; Supplementary Table 1). Each dimension is defined by one to eight indicators depending on the characteristics of the dimension: For instance, living standards include water, sanitation, energy, assets, housing, and livestock. Alkire and Foster (2011) developed the “dual cut-off method.” An individual is dimensionally poor if they fall below a threshold—the first cut-off—on any given indicator of a dimension of deprivation. An individual is multidimensionally poor when they are classified as poor on a contextually determined number of dimensions (Alkire & Foster, 2011). This second cut-off is set between one dimension, when one is considered poor if deprived of a unique dimension, and all dimensions (Bourguignon & Chakravarty, 2003; Deutsch & Silber, 2005; Tsui, 2002).

The first dimension of deprivation is education attainment. Literature shows education level influences health status through better hygiene and lifestyle, better work, and better economic conditions (Le & Nguyen, 2020; Ross & Wu, 1995; Vogl, 2012). Dimensional poverty was defined by the absence of enrollment in primary school. Existing data in Pakistan indicate that 44% of school-going children (5–16 years old), particularly girls and disadvantaged children are out of school (UNICEF, 2022).

Deprivation of health status was assessed by “a lot of difficulty in” or “cannot do at all” any of the 14 activities included in the short version of the Disability Screening Questionnaire (Trani et al., 2015). These limitations determine impairment that has been shown to be associated with multidimensional poverty in Pakistan (Mitra et al., 2013).

Eight indicators of deprivation of living standards were defined as crowded living space, unsafe access to water, cooking, heating, lighting sources, unimproved sanitation, owning fewer than six assets, or having no livestock. The different indicators’ cut-off determining deprivation were based on existing guidelines defined by United Nations Organizations, particularly UNICEF and WHO (see Table 1; WHO, 2018).

**Table 1.** Dimensions of Poverty and Indicators of Deprivation

Dimensions	Indicators	Deprivation cut-off (Deprived if … )
Education		
1. Adult educational attainment	What is your highest education level?	Did not go to school
Health status		
2. Activities limitation and functioning problem	Any difficulties you may have doing certain activities because of a health problem?	Participant answered “a lot of difficulty/cannot do at all” in ANY of the 14 questions of Disability Screening Questionnaire
Household-level material well-being/living standards		
3. Crowded space	How many people per room in your house?	More than three people per room
4. Water source	What is the main source of drinking water for your current household?	The household does not have “Piped drinking water in dwelling” or “Piped drinking water on site” or “Public tap/hand pump” or “Water carrier/tanker” or “Borehole on site/Hand pump on site” or “Borehole off site/communal” or “Covered well” or “Bottled water”
5. Energy source cooking	What is the main source of energy used by your household for cooking?	Deprived if the household does NOT use gas, solar energy or electricity instead uses wood, coal.
6. Energy source heating	What is the main source of energy used by your household for heating?	Deprived if the household does NOT use gas, solar energy or electricity instead uses wood, coal, other source such as plastic
7. Energy source lighting	What is the main source of energy used by your household for lighting?	Deprived if the household does NOT use gas, solar energy or electricity instead uses paraffin, wood, coal, candles, animal dung.
8. Sanitation	What type of toilet does your current household use?	The household does not “Own flush toilet inside the house” OR “Own flush toilet outside the house” or “Own Flush toilet or improved ventilated pit”
9. What assets does the household own?	Does any member of your household own any of the following? Radio, tape recorder/television/mobile phone/ pressure cooker–big pots/ refrigerator/ generator solar panels/bicycle–motorbike/car–tractor	Less than six assets. But if family own a tractor or a car they are automatically set as non-deprived
10. What livestock does your household own?	Does any member of your household own any of the following livestock? Camels/cows and buffalos/horse/donkeys/sheeps–goats/poultry	Owning no livestock
Economic activity		
11. Employment	Which one of the following best describes what you are currently doing? Are you currently looking for job and reason not looking for job	Deprived on employment if participant said “yes to currently looking for a job” OR “Participant was not looking for a job because he/she has the following reason: I became discouraged, I could not afford the cost of looking for work, The wages were too low”
11.B. Employment (adapted version)	Which one of the following best describes what you are currently doing? Are you currently looking for job and reason not looking for job?	Deprived on employment is decided on case by case, depends on the individual situation. Example, if participant answered not looking for a job because he/she is too old to work, we look at the participant’s age (below or above 60 years old), health status condition and current employment condition. Older adults with disability may have been willing to work but there is no job opportunity for them.
Social participation		
12. Discrimination and stigma (Experienced unfair treatment)	Have you been unfairly treated?	Stigma score is >1.5 (had at least moderate discrimination) on the unfair treatment scale.
Psychological well-being		
13. Depression	Center for Epidemiologic Studies—Depression Scale (CES-D). CES-D-10 scale is a self-report measure composed of 10 items assessing symptoms of depression in the last week. The total score—between 0 and 30—is calculated by adding the 10 items. Intensity of depression rises with the score with 10 being mild depression.	Depression score is ≥15 (moderate depression)
14. Distress	20-item Self-Reporting Questionnaire scale.	Distressed score is ≥9
15. Exposure to traumatic events	0–1 is minimal discrimination; 1–1.5 is low discrimination; 1.5–2 is moderate discrimination; and 2 and above is considered high discrimination	Exposure to five or more traumatic events.

Deprivation of employment has been shown to be an important economic indicator of multidimensional poverty (Basu & Nolen, 2008). In Pakistan, economic poverty is correlated to low productivity jobs in agriculture, a structurally low labor force participation, particularly for women (World Bank, 2023). Cut-off was defined as unemployment considering for a job or being discouraged from looking for a job because of low wage pay, old age, and having a disease or an impairment.

Deprivation of social participation was identified by moderate discrimination on the 22-item Unfair Treatment subscale of the Discrimination and Stigma Scale (Brohan et al., 2013). Discrimination and stigma have been shown to affect the quality of life and agency in various life domains, particularly for older adults (Walsh et al., 2017).

Finally, measures of depression, distress, and exposure to traumatic events defined deprivation of psychological well-being (Lund et al., 2010). Depression measured using the 10-item Center for Epidemiologic Studies Depression Scale Revised (CES-D-R-10; Radloff, 1977) has been linked to multidimensional poverty among older adults (Trani et al., 2022). A threshold of 15 was retained as the cut-off for moderate depression. Distress was assessed using the 20-item Self-Reporting Questionnaire (SRQ-20) after cultural adaptation (Rahman et al., 2003). The SRQ-20 has been used before to assess distress in the context of a different social group in Pakistan and a score of 9 and above has been found to be a valid cut-off for psychological problems (Rahman et al., 2009). The traumatic event checklist was used for the first time in Pakistan. We found a good scale reliability with Cronbach’s alpha of 0.93.

### Statistical Analysis

We measured the level of deprivation for older adults with and without dementia on each of the 15 indicators (Alkire & Foster, 2011). We then calculated multidimensional poverty measures considering two cut-offs, one within each dimension and one across dimensions: older adults were considered deprived in a given dimension if they fell below the dimensional cut-off and multidimensionally poor if they were deprived on at least d dimensions, with 1 < *d* < 15.

After checking for the normal distribution of the average deprivation share (*A*) using Q–Q plots, we calculated Students’ *t*-tests to confirm significant mean differences in (*A*) between older adults with RUDAS cut-offs of no dementia, mild dementia, or moderate-to-severe dementia stratifying by sex and age group (Ruxton & Beauchamp, 2008). We examined the overlap of indicators of deprivation using correlation analysis.

Logistic regression models examined the association between multidimensional poverty for *d* ≥ 4 and a binary outcome of either “no-to-mild” and “moderate-to-severe” dementia. This threshold corresponds prevalence of poverty of 13.2% among all older adults and 32.7% among older adults with dementia, comparable to the national poverty line established in 2018 at 21.9% of the population (World Bank, 2023). We adjusted the model for sex (male/female), age (continuous), marital status (living alone/with partner), and household size (continuous). A *p* value of <.05 was considered significant. Data were analyzed using Stata (v.16.1).

## Results

### Participant Characteristics

**Figure 1.**
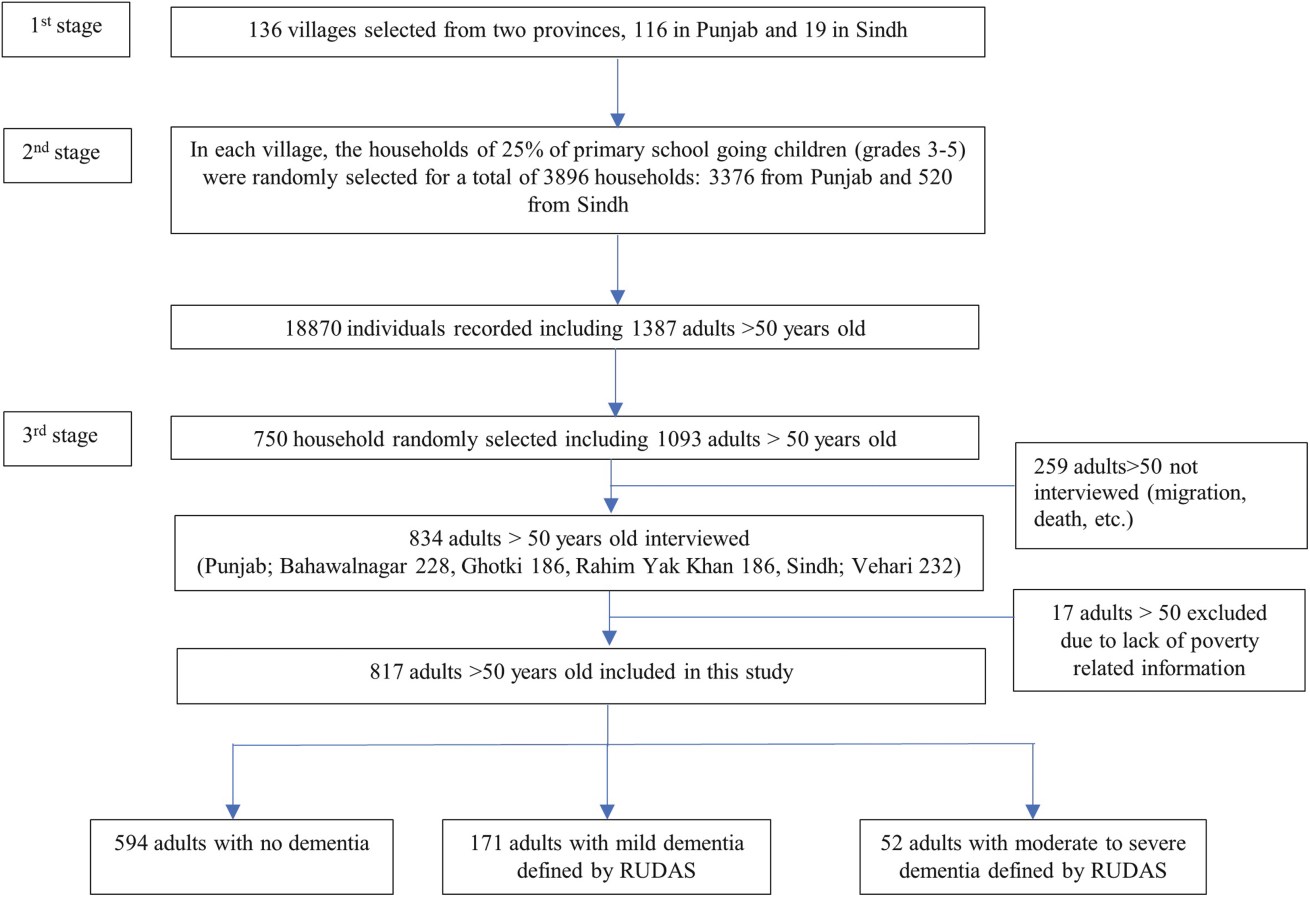
Participant recruitment through three-stage sampling selection. At the first stage of sampling, villages are selected. At the second stage, households within each village were randomly selected. At the third stage of sampling, older adults 50 years old or more were randomly selected among all adults 50 years old or more identified in the selected households.

From 1,093 available adults, we interviewed 834 older adults and excluded 259 (24%) due to attrition at the time of interview (death or migration, see Figure 1). An additional 17 (1.5%) older adults were excluded due to lack of poverty-related information. We identified 594 adults without dementia, 171 adults with mild dementia, and 52 with moderate-to-severe dementia using the RUDAS (see Table 2). A higher proportion of males (65.5%) compared to females (34.5%) showed no sign of dementia, whereas among adults with moderate-to-severe signs of dementia, 73.1% were female and only 26.9% were male (*p* < .001). Signs increased with age, and a higher proportion of adults above 70 had moderate-to-severe dementia (44.2%) compared to the age group 50–59 years old (23.1%, *p* < .001). A significantly higher proportion of older adults with moderate-to-severe dementia were living alone without partner (48.1%) compared to those without dementia (10.8%, *p* < .001).

**Table 2.** Sample Characteristics

Individual characteristics	No dementia	Mild dementia	Moderate-to-severe dementia	*p* Value
Gender (*n*, %)				
Male	389 (65.49)	82 (47.95)	14 (26.92)	<.001
Female	205 (34.51)	89 (52.05)	38 (73.08)	
Age (*n*, %)				
50–59	356 (59.93)	75 (43.86)	12 (23.08)	<.001
60–69	174 (29.29)	55 (32.16)	17 (32.69)	
≥70	64 (10.77)	41 (23.98)	23 (44.23)	
Age (mean, *SD*)	58.06 (7.42)	61.75 (9.50)	68.92 (12.06)	
Marital Status (*n*, %)				
Living with partner	530 (89.32)	132 (77.19)	27 (51.92)	<.001
Living alone	64 (10.77)	39 (22.81)	25 (48.08)	
Household size (mean, *SD*)	7.70 (2.79)	9.25 (3.65)	8.71 (3.50)	

*Note*: *SD* = standard deviation.

Older adults with moderate-to-severe dementia were significantly more deprived than those without dementia on 10 out of 15 indicators including education (96.2% vs 77.8%, *p* < .001), health (40.4% vs 18.4%, *p* < .001), cooking source (100% vs 90.2%, *p* = .048), lighting source (5.8% vs 1.9%, *p* = .011), sanitation (17.3% vs 10.6%, *p* = .045), assets (48.1% vs 15.0%, *p* < .001), stigma level (9.6% vs 3.2%, *p* = .01), depression level (55.8% vs 12.5%, *p* < .001), and distress level (63.4% vs 18.7%, *p* < .001). Remarkably, greater dementia severity was not associated with higher unemployment. Almost no participant was deprived of water or living in a crowded space. A higher proportion of male with moderate/severe dementia was significantly deprived on six indicators, including health (42.9% vs 15.7%, *p* = .008), lighting source (14.3% vs 2.1%, *p* = .003), sanitation (28.6% vs 11.3%, *p* = .01), assets (64.3% vs 14.9%, *p* < .001), depression (50.0% vs 10.3%, *p* < .001), and distress (57.1% vs 15.7%, *p* < .001) compared to those without dementia. More female with moderate/severe dementia than without dementia were significantly deprived of education (97.4% vs 93.7%, *p* = .04), assets (42.1% vs 15.1%, *p* < .001), and feel more depressed (57.9% vs 16.6%, *p* < .001) and distressed (65.8% vs 24.4%, *p* < .001). Sex differences on other indicators of deprivation were only marginally significant. More adults with dementia at age 50 and older were significantly deprived of assets and felt depressed and distressed.

There was limited association between indicators following Spearman rank correlation coefficient estimations (Table 3). Some living standards (sources of energy, assets, and sanitation), as well as mental well-being (depression, distress, trauma) indicators, were significantly but not strongly correlated with each other. We assigned each indicator an equal proportion of the dimensional weight (e.g., one eighth for each of the eight living standard indicators) in the equal nested weights structure to account for their proximity within the dimension (Alkire et al., 2015). Stigma was also correlated with depression, distress, and trauma, indicating how social exclusion and mental well-being are linked. Stigma shows how prejudice resulting from negative stereotypes translates into discrimination against a specific group, and is kept in a separate dimension (Link & Phelan, 2001). Limited correlations between indicators substantiate a multidimensional approach to poverty rather than a single welfare indicator such as income to represent complexity that innervates poverty.

**Table 3.** Spearman Rank Correlations Between Dimensions of Deprivation

Indicators of deprivation	1	2	3	4	5	6	7	8	9	10	11	12	13	14	15
1.School enrollment	1														
2.Difficulty functioning	0.0336	1													
3.Crowded space	−0.0115	0.0492	1												
4.Unsafe water	−0.1036^*^	0.0347	−0.0035	1											
5.Unsafe cooking source	0.0870^*^	0.0225	0.0216	0.0153	1										
6.Unsafe heating source	−0.0018	−0.0058	0.0117	0.0082	0.1366^*^	1									
7.Unsafe source of lighting	0.0087	0.0334	−0.0122	0.1381^*^	0.0537	0.0289	1								
8.Poor sanitation	0.0362	0.0862^*^	−0.0263	0.0566	0.0763^*^	−0.0294	0.3311^*^	1							
9.Lack of assets	0.0879^*^	−0.0038	−0.0349	0.0375	0.1316^*^	0.0069	0.1680^*^	0.2049^*^	1						
10.No livestock	−0.0526	−0.0283	−0.0282	−0.0199	−0.0513	−0.0203	−0.0701^*^	−0.0332	−0.0231	1					
11.Unemployment	−0.0067	0.1376^*^	0.0544	−0.0128	−0.0102	0.0429	0.015	−0.0201	−0.0141	0.0129	1				
12.Stigma	0.0739^*^	0.1024^*^	−0.0153	−0.0108	−0.0373	−0.0001	−0.003	−0.0639	0.0984^*^	−0.0537	−0.0319	1			
13.Depression	0.1042^*^	0.1352^*^	0.0105	−0.0241	0.0393	0.0423	0.0261	0.0351	0.1802^*^	−0.0698^*^	0.0291	0.1787^*^	1		
14.Distress	0.0911^*^	0.0893^*^	−0.0369	−0.0261	0.0251	0.0324	0.0669	0.0643	0.1409^*^	−0.0574	0.0608	0.2284^*^	0.6290^*^	1	
15.Trauma	−0.0005	0.0344	−0.0099	−0.007	0.0436	0.0235	−0.0246	−0.0262	−0.0481	−0.0314	0	0.1391^*^	0.0662	0.1187^*^	1

^*^ *p* < .05.

### Multidimensional Poverty

The poverty headcount (*H*) for a cut-off *d* = 1 (union approach; Bourguignon & Chakravarty, 2003) showed that, respectively, 84.2%, 95.9%, and 100% of adults with no, mild, and moderate-to-severe dementia were poor on at least one dimension (Supplementary Table 2). Implementing the intersectional approach (*d* = 7) found that 0.4% were poor in all dimensions. A higher number of male and female with moderate-to-severe dementia were poorer irrespective of the number of dimensions compared to, respectively, male and female without dementia (for *d* ≤ 6). More female with moderate-to-severe dementia were poorer on 1 ≤ *d* = <3 compared to male with moderate-to-severe dementia, but the reverse was true for *d* > 3. The proportion of poor adults without dementia was lower at 13.2% compared to 21.6% and 32.7%, respectively, among those with mild and moderate-to-severe dementia (for *d* = 4).

**Figure 2.**
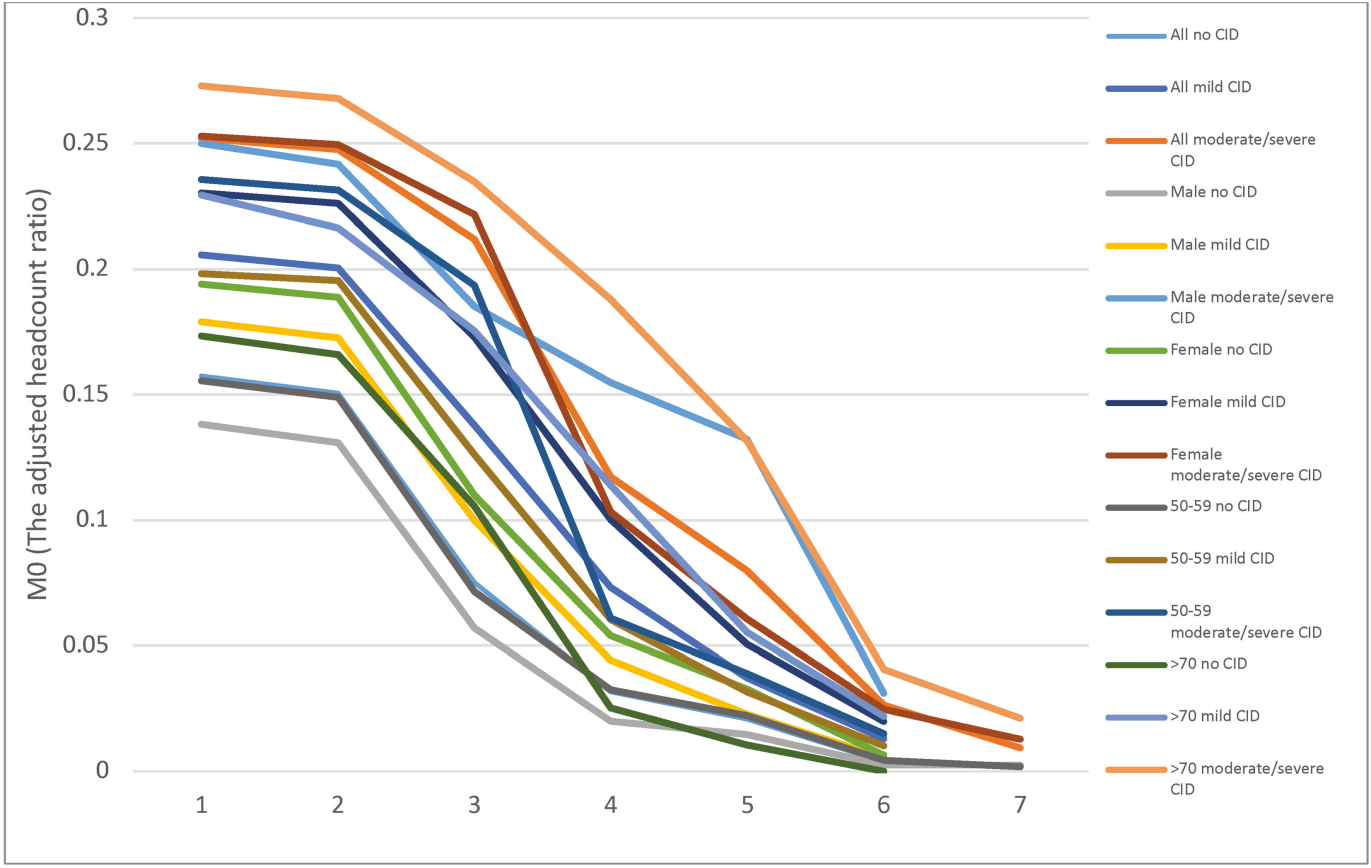
Adjusted poverty headcount equal nested weighting structure by dementia status and by sex and age group.

In terms of intensity or depth of poverty, the adjusted headcount ratio (*M*0) was significantly higher for older adults with moderate-to-severe dementia compared to those without dementia whatever the cut-off *d-*value between one and six with a difference varying between 60.1% for *d* = 1 and 585.3% for *d* = 6 (see Figure 2 and Supplementary Table 3). Depth of poverty was significantly greater whatever the threshold (*d*) for both female and male with moderate-to-severe dementia compared, respectively, to female and male without dementia (see Figure 2 and Supplementary Table 4). For *d* = 4, the gender difference in depth of poverty according to level of dementia is, respectively, 670.8% (*p* < .001) and 91.1% (*p* = .038) between male and female with moderate-to-severe dementia compared to those without dementia. The depth of multidimensional poverty was significantly greater among adults older than 70 years old with moderate-to-severe dementia for 1 < *d* < 7 with a difference of 646.8% for *d *= 4 (*p* < .001). This difference was significant for *d* < 5 for adults aged 50–69 years old (Supplementary Table 5).

Education was the highest contributor to multidimensional poverty regardless of the cut-off *d* whatever the dementia status, for both sex and age groups (see Table 4). Interestingly, health was the second contributor to poverty for all older adults, whatever their characteristics for *d* ≥ 3; while it was living standards for *d* < 3, except in the case of older adults over 70 years old for whom health was the second contributor whatever the dementia status. Deprivation of employment was a higher contributor than living standards for older adults of both sexes but only for those without dementia and *d* > 3. Instead, psychological well-being was the third contributor to poverty, before living standards, whatever the cut-off *d* for women—or adults over 70 years old—both with dementia, but only for *d* > 2 for male—or adults 50–69 years old—with dementia. Stigma contributed more than 10% to multidimensional poverty only for older adults over 70 years old with dementia and for *d* > 2.

**Table 4.** Percentage Contribution of Each Dimension to Multidimensional Poverty by Dementia Status and by Sex and Age Group

Number of dimensions of deprivation	All	All			Male			Female			Age 50–69			Over 70		
		No CID	Mild CID	Moderate/severe CID	No CID	Mild CID	Moderate/severe CID	No CID	Mild CID	Moderate/severe CID	No CID	Mild CID	Moderate/severe CID	No CID	Mild CID	Moderate/severe CID
Dimension 1																
Education	0.529	0.557	0.493	0.429	0.567	0.489	0.417	0.544	0.496	0.433	0.560	0.511	0.455	0.533	0.444	0.399
Health	0.139	0.131	0.142	0.180	0.128	0.153	0.193	0.136	0.134	0.175	0.119	0.128	0.179	0.225	0.180	0.181
Living conditions	0.171	0.000	0.168	0.154	0.184	0.192	0.176	0.160	0.151	0.146	0.175	0.169	0.171	0.160	0.168	0.136
Employment status	0.041	0.000	0.029	0.026	0.038	0.023	0.032	0.059	0.033	0.023	0.053	0.040	0.049	0.000	0.000	0.000
Stigma	0.029	0.068	0.042	0.043	0.019	0.023	0.032	0.028	0.056	0.047	0.026	0.035	0.000	0.000	0.060	0.091
Psychosocial well-being	0.092	0.072	0.126	0.169	0.064	0.120	0.150	0.073	0.130	0.175	0.066	0.118	0.146	0.082	0.148	0.193
Dimension 2																
Education	0.525	0.002	0.492	0.428	0.564	0.500	0.432	0.535	0.486	0.427	0.555	0.508	0.447	0.520	0.446	0.406
Health	0.142	0.009	0.146	0.183	0.130	0.159	0.199	0.140	0.137	0.178	0.121	0.130	0.182	0.233	0.191	0.185
Living conditions	0.171	0.012	0.166	0.153	0.185	0.185	0.170	0.161	0.152	0.147	0.177	0.169	0.172	0.162	0.159	0.132
Employment status	0.042	0.011	0.030	0.026	0.040	0.024	0.033	0.061	0.034	0.024	0.056	0.040	0.050	0.000	0.000	0.000
Stigma	0.031	0.047	0.043	0.044	0.020	0.024	0.033	0.029	0.057	0.047	0.027	0.036	0.000	0.000	0.064	0.092
Psychosocial well-being	0.090	0.023	0.123	0.166	0.061	0.108	0.133	0.073	0.133	0.178	0.064	0.117	0.149	0.085	0.140	0.185
Dimension 3																
Education	0.382	0.030	0.379	0.372	0.384	0.359	0.348	0.388	0.389	0.380	0.381	0.388	0.393	0.411	0.358	0.350
Health	0.234	0.034	0.208	0.230	0.258	0.239	0.261	0.241	0.191	0.219	0.232	0.187	0.240	0.343	0.256	0.219
Living conditions	0.126	0.004	0.130	0.133	0.129	0.142	0.130	0.116	0.123	0.133	0.122	0.127	0.148	0.126	0.136	0.118
Employment status	0.067	0.551	0.047	0.022	0.071	0.045	0.043	0.105	0.048	0.015	0.105	0.067	0.044	0.000	0.000	0.000
Stigma	0.055	0.135	0.067	0.055	0.049	0.045	0.043	0.047	0.079	0.058	0.057	0.060	0.000	0.000	0.085	0.109
Psychosocial well-being	0.136	0.000	0.170	0.190	0.108	0.170	0.174	0.103	0.170	0.195	0.103	0.172	0.175	0.120	0.165	0.204
Dimension 4																
Education	0.325	0.000	0.334	0.327	0.298	0.331	0.313	0.330	0.335	0.332	0.311	0.333	0.341	0.368	0.336	0.316
Health	0.251	0.070	0.235	0.296	0.266	0.271	0.313	0.230	0.219	0.288	0.228	0.203	0.341	0.368	0.288	0.264
Living conditions	0.108	0.071	0.115	0.119	0.096	0.128	0.130	0.103	0.110	0.114	0.099	0.112	0.128	0.105	0.120	0.112
Employment status	0.089	0.002	0.072	0.016	0.141	0.090	0.052	0.120	0.065	0.000	0.145	0.116	0.038	0.000	0.000	0.000
Stigma	0.089	0.009	0.099	0.078	0.094	0.090	0.052	0.080	0.103	0.089	0.097	0.087	0.000	0.000	0.120	0.132
Psychosocial well-being	0.139	0.012	0.144	0.166	0.105	0.090	0.139	0.137	0.168	0.177	0.120	0.150	0.152	0.158	0.136	0.176
Dimension 5																
Education	0.293	0.011	0.284	0.297	0.296	0.280	0.300	0.295	0.287	0.294	0.294	0.285	0.296	0.329	0.284	0.297
Health	0.239	0.049	0.197	0.297	0.237	0.224	0.300	0.236	0.179	0.294	0.234	0.178	0.296	0.329	0.227	0.297
Living conditions	0.101	0.024	0.096	0.118	0.099	0.084	0.120	0.096	0.103	0.117	0.096	0.098	0.111	0.123	0.092	0.121
Employment status	0.125	0.029	0.109	0.027	0.178	0.168	0.060	0.157	0.072	0.000	0.173	0.178	0.099	0.000	0.000	0.000
Stigma	0.109	0.033	0.153	0.081	0.099	0.112	0.060	0.098	0.179	0.098	0.102	0.107	0.000	0.000	0.227	0.111
Psychosocial well-being	0.134	0.005	0.160	0.180	0.092	0.131	0.160	0.118	0.179	0.196	0.102	0.154	0.198	0.219	0.170	0.173
Dimension 6																
Education	0.248	0.386	0.249	0.250	0.232	0.247	0.255	0.258	0.250	0.247	0.244	0.251	0.255	0.000	0.247	0.247
Health	0.248	0.249	0.249	0.250	0.232	0.247	0.255	0.258	0.250	0.247	0.244	0.251	0.255	0.000	0.247	0.247
Living conditions	0.084	0.000	0.086	0.083	0.072	0.093	0.064	0.097	0.083	0.093	0.084	0.079	0.064	0.000	0.093	0.093
Employment status	0.068	0.000	0.000	0.083	0.116	0.000	0.255	0.129	0.000	0.000	0.122	0.000	0.255	0.000	0.000	0.000
Stigma	0.203	0.046	0.249	0.167	0.232	0.247	0.000	0.129	0.250	0.247	0.183	0.251	0.000	0.000	0.247	0.247
Psychosocial well-being	0.150	0.049	0.166	0.167	0.116	0.165	0.170	0.129	0.167	0.165	0.122	0.168	0.170	0.000	0.165	0.165
Dimension 7																
Education	0.232	0.001	0.000	0.000	0.232	0.000	0.000	0.000	0.000	0.000	0.232	0.000	0.000	0.000	0.000	0.000
Health	0.232	0.008	0.000	0.000	0.232	0.000	0.000	0.000	0.000	0.000	0.232	0.000	0.000	0.000	0.000	0.000
Living conditions	0.072	0.010	0.000	0.000	0.072	0.000	0.000	0.000	0.000	0.000	0.072	0.000	0.000	0.000	0.000	0.000
Employment status	0.116	0.007	0.000	0.000	0.116	0.000	0.000	0.000	0.000	0.000	0.116	0.000	0.000	0.000	0.000	0.000
Stigma	0.232	0.088	0.000	0.000	0.232	0.000	0.000	0.000	0.000	0.000	0.232	0.000	0.000	0.000	0.000	0.000
Psychosocial well-being	0.116	0.048	0.000	0.000	0.116	0.000	0.000	0.000	0.000	0.000	0.116	0.000	0.000	0.000	0.000	0.000

*Note:* CID stands for cognitive impairment and dementia.

### Multivariate Analysis

The risk of dementia was 8.59 (95% CI, 3.89–18.92) times higher for multidimensionally poor older adults, for a cut-off *d* = 4. It was 3.88 times higher (95% CI, 1.61–9.31) for poor older adults adjusting for sex, marital status, age, and household size. It was even higher for adults ≥70 years (65.5, 95% CI, 6.87–624.24, see Table 5). Being female significantly increased the relative risk of dementia by 2.51 (95% CI, 1.83–3.43, *p* < .001) in the unadjusted model and by 2.07 (95% CI, 1.44–2.95, *p* < .001) in the adjusted model but not significantly if we only consider adults >70 years old. Similarly, living without a partner increased the risk for older adults in the unadjusted (3.33, 95% CI, 2.25-4.91, *p* < .001) and adjusted models (1.84, 95% CI, 1.16–2.92, *p* = .009), but not significantly if we only consider adults >70 years old. Household size also significantly increased the risk in all models.

**Table 5.** Logistic Regression Results of Poverty on Dementia

Independent variables	Unadjusted analysis			Adjusted analysis 50 and over			Adjusted analysis 70 and over		
	OR	(95% CI)	*p* Value	OR	(95% CI)	*p* Value	OR	(95% CI)	*p* Value
Multidimensionally poor (Ref: not poor)	8.59	3.89–18.92	<.001	3.88	1.61–9.31	.002	65.5	6.87–624.2	<.001
Female (Ref: male)	2.51	1.83–3.43	<.001	2.07	1.44–2.95	<.001	1.11	0.45–2.71	.814
Living alone (Ref: living with partner)	3.33	2.25–4.91	<.001	1.84	1.16–2.92	.009	2.15	0.85–5.42	.105
Age (continuous)	1.07	1.05–1.08	<.001	1.06	1.03–1.07	<.001	1.08	1.00–1.16	.03
Household size (continuous)	1.15	1.09–1.212	<.001	1.16	1.10–1.22	<.001	1.25	1.07–1.46	.005
Constant				0.00	0.00–0.00	<.001	0	0.00–0.05	.003

*Notes*: CI = confidence interval; OR = odd ratio; Ref = reference category. The model was fully adjusted with sex, marital status, age, and household size.

### Sensitivity Analysis

Multidimensional poverty calculation was repeated using the equal-indicator weight structure between the six dimensions and results were held. Multidimensional poverty was found to be significantly higher for older adults with moderate-to-severe dementia compared to older adults without dementia for any cut-off *d*. Both women and men with dementia were poorer than women and men without dementia for all *d*. Similarly, the depth of poverty was higher for adults over 70 and adults 50–69 years old with dementia compared to those without dementia. Contributions of dimensions to *(M*0) consistently showed the pre-eminence of education and health for older adults with and without dementia and for each sex.

## Discussion and Implications

Our study contributes to a small but critically emerging body of research that investigates the intersection of social and environmental determinants of health (SEDOH) on dementia in high (Hofbauer & Rodriguez, 2021, 2023; Vega et al., 2017) and LMICs (Gurukartick et al., 2016). It is well established that poor SEDOH are associated with overall poorer health outcomes (Marmot et al., 2008). Overlapping scientific priorities from neurology and human development has centralized SEDOH to better understand dementia risk. This is further justified by the fact that many of the 12 identified modifiable risk factors are SEDOH (Livingston et al., 2020) and particularly those linked to different facets of deprivation: low standards in diet, clothing, housing, and household facilities, but also access to employment, health care, and social activities (Townsend, 1987). Our approach is unique in its operationalization of this complex concept of deprivation and the examination of its link with cognitive decline. We used a set of measures of multidimensional poverty in six domains of SEDOH—education, health status, living standards, economic activity, social participation, and psychological well-being to demonstrate their combined impact on dementia.

Findings show that adults above 50 years old with dementia, particularly women and those older, were deprived in more domains compared to adults without dementia. Similarly, the association was stronger among adults older than 70 years old. Higher association of severe poverty with dementia among women and increasing with age has already been shown in Afghanistan (Trani et al., 2023), Iran (Mohaqeqi Kamal et al., 2022), and South Africa (Trani et al., 2022) using a similar approach and confirms how gender inequity set women on a different life path resulting in greater dementia risk for them in LMICs (Mielke et al., 2022).

Education and health status contributed the most to multidimensional poverty for all adults, confirming earlier evidence that quality and free education and health care services earlier in life, but also during the life cycle are necessary to not only promote well-being and equity but also to reduce dementia risk at a later age (WHO, 2017). Psychological well-being was also a major contributor to multidimensional poverty for women with dementia, and adults over 70 years old with dementia of both sexes, adding to an abundant literature that already established a link between poverty and poor mental health (Lund et al., 2010) on one hand and the latter with the risk of dementia on the other hand (Babulal et al., 2022; Hofbauer & Rodriguez, 2023). Interestingly, access to employment was a major contributor to poverty for older adults without dementia only, indicating that working was not expected any more from older adults with dementia in Pakistan whereas other older adults need to work, a finding that warrants further investigation (Jalal & Younis, 2014).

Conversely, stigma contributed to poverty for adults over 70 years with dementia confirming prior studies that evince links between discrimination fueled by different characteristics (gender, race, ethnicity, socioeconomic status, sexual orientation), chronic stress, poorer access and quality of care, and earlier onset of age-related diseases such as dementia (Carroll et al., 2022; Fam et al., 2019; Geronimus et al., 2015). Living with a partner was a protective factor for dementia, showing the importance of social and emotional support in mediating the deleterious pathophysiological effects of aging. Belonging to a large household increased the risk of dementia, which may be explained by a heightened burden of poverty common to larger households with less support and resources available to older members (Tellez et al., 2016).

### Limitations

Our study presents with a few limitations. The cross-sectional nature of our data does not allow to establish a direction for causality between poverty and dementia. Second, the RUDAS established various cognitive limitations but is unfit to determine a formal diagnosis by a physician and which would require corroboration by conventional biomarkers (e.g., imaging cerebrospinal fluid). Third, sample size was too small to further investigate the association between multidimensional poverty and dementia beyond 75 years old. Fourth, our sample is limited to some districts of Punjab and Sindh and cannot be generalized to Pakistan altogether. Finally, 25.3% of participants in the initial sample were missing interviews, which might have introduced bias in our results because we cannot assume they were missing completely at random.

Our findings emphasize the role of socioeconomic justice in advocating for public policies to address enduring sources of detrimental health outcomes. They corroborate existing evidence showing the importance of free universal and quality education and health care and suggest that similar mechanisms linking poverty to cognitive status in old age exist across LMICs. Further research should investigate this link in other social contexts. Physicians and public health professionals must consider dementia as a public health priority, advocate for early intervention on social determinants of health to prevent dementia, and promote social care to reduce the current burden of the disease currently shouldered almost entirely by people with dementia and their families in LMICs.

## Supplementary Material

igae007_suppl_Supplementary_Tables_S1-S5
