# Supplementary material for: Is Multidimensional Poverty Associated to Dementia Risk? The Case of Older Adults in Pakistan
Source: Innov Aging. 2024 Feb 1;8(2):igae007. doi: 10.1093/geroni/igae007 (PMC10924444; doi:10.1093/geroni/igae007)
Supplement: igae007_suppl_Supplementary_Tables_S1-S5 [file igae007_suppl_supplementary_tables_s1-s5.docx]

***Innovation in Aging* Supplementary Material: Jean- François Trani, Yiqi Zhu, Soobin Park, & Ganesh M. Babulal. Is multidimensional poverty associated to dementia risk? The case of older adults in Pakistan.**

# Supplementary Material

# Supplementary Table 1. Proportion of participants deprived in each dimension by status of dementia and sex.

| Indicator | All | | | | Male | | | | Female | | | |
| --- | --- | --- | --- | --- | --- | --- | --- | --- | --- | --- | --- | --- |
|  | No dementia | Mild dementia | Moderate to severe dementia | P Value | No dementia | Mild dementia | Moderate to severe dementia | P Value | No dementia | Mild dementia | Moderate to severe dementia | P Value |
| School enrolment | 462 (77.78) | 153 (89.47) | 50 (96.15) | <0.001 | 270 (69.41) | 64 (78.05) | 13 (92.86) | 0.058 | 192 (93.66) | 89 (100) | 37 (97.37) | 0.04 |
| Difficulty functioning | 109 (18.35) | 44 (25.73) | 21 (40.38) | <0.001 | 61 (15.68) | 20 (24.39) | 6 (42.86) | 0.008 | 48 (23.41) | 24 (26.97) | 15 (39.47) | 0.116 |
| Crowded space | 1 (0.17) | 3 (1.75) | 0 (0) | 0.028 | 1 (0.26) | 1 (1.22) | 0 (0) | 0.452 | 0 (0) | 2 (2.25) | 0 (0) | 0.064 |
| Unsafe water | 1 (0.17) | 1 (0.58) | 0 (0) | 0.583 | 1 (0.26) | 1 (1.22) | 0 (0) | 0.452 | 0 (0) | 0 (0) | 0 (0) | n/a |
| Unsafe cooking source | 536 (90.24) | 158 (92.4) | 52 (100) | 0.048 | 356 (91.52) | 77 (93.9) | 14 (100) | 0.415 | 180 (87.8) | 81 (91.01) | 38 (100) | 0.066 |
| Unsafe heating source | 574 (96.63) | 170 (99.42) | 51 (98.08) | 0.132 | 375 (96.4) | 82 (100) | 14 (100) | 0.169 | 199 (97.07) | 88 (98.88) | 37 (97.37) | 0.648 |
| Unsafe source of lighting | 11 (1.85) | 10 (5.85) | 3 (5.77) | 0.011 | 8 (2.06) | 6 (7.32) | 2 (14.29) | 0.003 | 3 (1.46) | 4 (4.49) | 1 (2.63) | 0.296 |
| Poor sanitation | 63 (10.61) | 29 (16.96) | 9 (17.31) | 0.045 | 44 (11.31) | 18 (21.95) | 4 (28.57) | 0.01 | 19 (9.27) | 11 (12.36) | 5 (13.16) | 0.625 |
| Lack of assets | 89 (14.98) | 48 (28.07) | 25 (48.08) | <0.001 | 58 (14.91) | 26 (31.71) | 9 (64.29) | <0.001 | 31 (15.12) | 22 (24.72) | 16 (42.11) | <0.001 |
| Lack of animals | 95 (15.99) | 15 (8.77) | 4 (7.69) | 0.023 | 58 (14.91) | 6 (7.32) | 1 (7.14) | 0.146 | 37 (18.05) | 9 (10.11) | 3 (7.89) | 0.095 |
| Unemployment | 39 (6.57) | 9 (5.26) | 3 (5.77) | 0.816 | 18 (4.63) | 3 (3.66) | 1 (7.14) | 0.83 | 21 (10.24) | 6 (6.74) | 2 (5.26) | 0.449 |
| Discrimination | 19 (3.2) | 13 (7.6) | 5 (9.62) | 0.01 | 9 (2.31) | 3 (3.66) | 1 (7.14) | 0.456 | 10 (4.88) | 10 (11.24) | 4 (10.53) | 0.109 |
| Depression | 74 (12.46) | 53 (30.99) | 29 (55.77) | <0.001 | 40 (10.28) | 23 (28.05) | 7 (50) | <0.001 | 34 (16.59) | 30 (33.71) | 22 (57.89) | <0.001 |
| Distress | 86 (14.48) | 62 (36.26) | 29 (55.77) | <0.001 | 47 (12.08) | 23 (28.05) | 7 (50) | <0.001 | 39 (19.02) | 39 (43.82) | 22 (57.89) | <0.001 |
| Trauma | 13 (2.19) | 2 (1.17) | 1 (1.92) | 0.698 | 9 (2.31) | 1 (1.22) | 0 (0) | 0.703 | 4 (1.95) | 1 (1.12) | 1 (2.63) | 0.817 |

# Supplementary Table 2. Proportion of participants deprived in each dimension by status of dementia and age group.

| Indicator | All | | | | 50-59 | | | | 60-69 | | | | 70-79 | | | |
| --- | --- | --- | --- | --- | --- | --- | --- | --- | --- | --- | --- | --- | --- | --- | --- | --- |
|  | No dementia | Mild dementia | Moderate to severe dementia | P Value | No dementia | Mild dementia | Moderate to severe dementia | P Value | No dementia | Mild dementia | Moderate to severe dementia | P Value | No dementia | Mild dementia | Moderate to severe dementia | P Value |
| School enrolment | 462 (77.78) | 153 (89.47) | 50 (96.15) | <0.001 | 267 (75) | 68 (90.67) | 12 (100) | 0.002 | 143 (82.18) | 48 (87.27) | 16 (94.12) | 0.338 | 52 (81.25) | 37 (90.24) | 22 (95.65) | 0.157 |
| Difficulty functioning | 109 (18.35) | 44 (25.73) | 21 (40.38) | <0.001 | 51 (14.33) | 14 (18.67) | 3 (25) | 0.41 | 36 (20.69) | 15 (27.27) | 8 (47.06) | 0.042 | 22 (34.38) | 15 (36.59) | 10 (43.48) | 0.739 |
| Crowded space | 1 (0.17) | 3 (1.75) | 0 (0) | 0.028 | 0 (0) | 1 (1.33) | 0 (0) | 0.086 | 0 (0) | 1 (1.82) | 0 (0) | 0.175 | 1 (1.56) | 1 (2.44) | 0 (0) | 0.752 |
| Unsafe water | 1 (0.17) | 1 (0.58) | 0 (0) | 0.583 | 1 (0.28) | 1 (1.33) | 0 (0) | 0.453 | 0 (0) | 0 (0) | 0 (0) | n/a | 0 (0) | 0 (0) | 0 (0) | n/a |
| Unsafe cooking source | 536 (90.24) | 158 (92.4) | 52 (100) | 0.048 | 321 (90.17) | 68 (90.67) | 12 (100) | 0.52 | 157 (90.23) | 52 (94.55) | 17 (100) | 0.265 | 58 (90.63) | 38 (92.68) | 23 (100) | 0.319 |
| Unsafe heating source | 574 (96.63) | 170 (99.42) | 51 (98.08) | 0.132 | 341 (95.79) | 75 (100) | 12 (100) | 0.15 | 169 (97.13) | 54 (98.18) | 16 (94.12) | 0.678 | 0 (0) | 0 (0) | 0 (0) | n/a |
| Unsafe source of lighting | 11 (1.85) | 10 (5.85) | 3 (5.77) | 0.011 | 6 (1.69) | 4 (5.33) | 2 (16.67) | 0.002 | 4 (2.3) | 2 (3.64) | 1 (5.88) | 0.644 | 1 (1.56) | 4 (9.76) | 0 (0) | 0.061 |
| Poor sanitation | 63 (10.61) | 29 (16.96) | 9 (17.31) | 0.045 | 38 (10.67) | 15 (20) | 4 (33.33) | 0.009 | 21 (12.07) | 6 (10.91) | 2 (11.76) | 0.973 | 4 (6.25) | 8 (19.51) | 3 (13.04) | 0.117 |
| Lack of assets | 89 (14.98) | 48 (28.07) | 25 (48.08) | <0.001 | 51 (14.33) | 19 (25.33) | 6 (50) | 0.001 | 28 (16.09) | 10 (18.18) | 10 (58.82) | <0.001 | 10 (15.63) | 19 (46.34) | 9 (39.13) | 0.002 |
| Lack of animals | 95 (15.99) | 15 (8.77) | 4 (7.69) | 0.023 | 59 (16.57) | 8 (10.67) | 1 (8.33) | 0.345 | 31 (17.82) | 6 (10.91) | 1 (5.88) | 0.246 | 5 (7.81) | 1 (2.44) | 2 (8.7) | 0.468 |
| Unemployment | 39 (6.57) | 9 (5.26) | 3 (5.77) | 0.816 | 21 (5.9) | 8 (10.67) | 2 (16.67) | 0.14 | 18 (10.34) | 1 (1.82) | 1 (5.88) | 0.123 | 0 (0) | 0 (0) | 0 (0) | n/a |
| Discrimination | 19 (3.2) | 13 (7.6) | 5 (9.62) | 0.01 | 13 (3.65) | 4 (5.33) | 0 (0) | 0.617 | 6 (3.45) | 4 (7.27) | 0 (0) | 0.31 | 0 (0) | 5 (12.2) | 5 (21.74) | 0.002 |
| Depression | 74 (12.46) | 53 (30.99) | 29 (55.77) | <0.001 | 35 (9.83) | 17 (22.67) | 6 (50) | <0.001 | 29 (16.67) | 17 (30.91) | 9 (52.94) | 0.001 | 10 (15.63) | 19 (46.34) | 14 (60.87) | <0.001 |
| Distress | 86 (14.48) | 62 (36.26) | 29 (55.77) | <0.001 | 48 (13.48) | 23 (30.67) | 6 (50) | <0.001 | 26 (14.94) | 21 (38.18) | 6 (35.29) | <0.001 | 12 (18.75) | 18 (43.9) | 17 (73.91) | <0.001 |
| Trauma | 13 (2.19) | 2 (1.17) | 1 (1.92) | 0.698 | 7 (1.97) | 1 (1.33) | 0 (0) | 0.832 | 2 (1.15) | 1 (1.82) | 0 (0) | 0.827 | 4 (6.25) | 0 (0) | 1 (4.35) | 0.27 |

**Supplementary Table 3 Multidimensional Poverty Measures for Persons with and without Dementia with equal nested weight**

|  | All | | | No CID | | | Mild CID | | | | |  | Moderate/severe CID | | | |
| --- | --- | --- | --- | --- | --- | --- | --- | --- | --- | --- | --- | --- | --- | --- | --- | --- |
| d | H | A | M0 | H | A | M0 | H | A | M0 | P value | % difference in M0 | H | A | M0 | P value | % difference in M0 |
| 1 | 0.876 | 0.198 | 0.174 | 0.842 | 0.187 | 0.157 | 0.959 | 0.214 | 0.206 | <.001 | 30.63 | 1 | 0.252 | 0.252 | <.001 | 60.12 |
| 2 | 0.823 | 0.203 | 0.167 | 0.785 | 0.192 | 0.151 | 0.912 | 0.220 | 0.200 | <.001 | 32.94 | 0.962 | 0.257 | 0.248 | <.001 | 64.17 |
| 3 | 0.348 | 0.279 | 0.097 | 0.271 | 0.278 | 0.075 | 0.497 | 0.278 | 0.138 | <.001 | 83.31 | 0.731 | 0.290 | 0.212 | <.001 | 181.58 |
| 4 | 0.132 | 0.347 | 0.046 | 0.091 | 0.350 | 0.032 | 0.216 | 0.339 | 0.073 | <.001 | 130.50 | 0.327 | 0.358 | 0.117 | <.001 | 268.35 |
| 5 | 0.075 | 0.376 | 0.028 | 0.056 | 0.376 | 0.021 | 0.099 | 0.376 | 0.037 | 0.042 | 78.87 | 0.212 | 0.378 | 0.080 | <.001 | 282.62 |
| 6 | 0.016 | 0.451 | 0.007 | 0.008 | 0.456 | 0.004 | 0.029 | 0.444 | 0.013 | 0.040 | 238.19 | 0.058 | 0.457 | 0.026 | 0.002 | 587.25 |
| 7 | 0.004 | 0.481 | 0.002 | 0.003 | 0.479 | 0.002 | 0.000 | NA | NA | NA | NA | 0.019 | 0.486 | 0.009 | 0.103 | 479.42 |

**Supplementary Table 4 Multidimensional Poverty Measures for Persons with and without Dementia by gender with equal nested weights**

| Male | | | | | | | | | | | | | | | | | | |
| --- | --- | --- | --- | --- | --- | --- | --- | --- | --- | --- | --- | --- | --- | --- | --- | --- | --- | --- |
|  | No CID | | | Mild CID | | | | | | Moderate to severe | | | | | | | | |
| d | H | A | Mo | H | A | Mo | P value | % difference in M0 | | H | | A | | Mo | P value | | % difference in M0 | |
| 1 | H | A | M0 | H | A | M0 | P value | % difference in M0 | | H | | A | | M0 | P value | | % difference in M0 | |
| 2 | 0.779 | 0.177 | 0.138 | 0.915 | 0.196 | 0.179 | <.001 | 29.56 | | 1 | | 0.250 | | 0.250 | <.001 | | 80.91 | |
| 3 | 0.715 | 0.183 | 0.131 | 0.854 | 0.202 | 0.173 | <.001 | 32.04 | | 0.929 | | 0.260 | | 0.242 | <.001 | | 84.93 | |
| 4 | 0.211 | 0.270 | 0.057 | 0.378 | 0.265 | 0.100 | 0.027 | 75.71 | | 0.571 | | 0.324 | | 0.185 | <.001 | | 225.25 | |
| 5 | 0.057 | 0.355 | 0.020 | 0.134 | 0.329 | 0.044 | 0.027 | 119.90 | | 0.429 | | 0.361 | | 0.155 | <.001 | | 670.82 | |
| 6 | 0.039 | 0.380 | 0.015 | 0.061 | 0.374 | 0.023 | 0.384 | 55.56 | | 0.357 | | 0.370 | | 0.132 | <.001 | | 802.13 | |
| 7 | 0.005 | 0.479 | 0.002 | 0.012 | 0.449 | 0.005 | 0.508 | 122.30 | | 0.071 | | 0.435 | | 0.031 | 0.084 | | 1161.76 | |
| Female | | | | | | | | | | | | | | | | | |  |
|  | No CID | | | Mild CID | | | | | | Moderate/severe CID | | | | | | | |  |
| d | H | A | Mo | H | A | Mo | P value | | % difference in M0 | H | A | | Mo | | | P value | % difference in M0 |  |
| 1 | 0.961 | 0.202 | 0.194 | 1 | 0.230 | 0.230 | 0.002 | | 18.67 | 1 | 0.253 | | 0.253 | | | <.001 | 30.33 |  |
| 2 | 0.917 | 0.206 | 0.189 | 0.966 | 0.234 | 0.226 | 0.006 | | 19.73 | 0.974 | 0.256 | | 0.250 | | | <.001 | 32.21 |  |
| 3 | 0.385 | 0.285 | 0.110 | 0.607 | 0.285 | 0.173 | 0.004 | | 57.14 | 0.789 | 0.281 | | 0.222 | | | <.001 | 101.57 |  |
| 4 | 0.156 | 0.346 | 0.054 | 0.292 | 0.343 | 0.100 | 0.013 | | 85.35 | 0.289 | 0.356 | | 0.103 | | | 0.038 | 91.11 |  |
| 5 | 0.088 | 0.372 | 0.033 | 0.135 | 0.376 | 0.051 | 0.581 | | 55.31 | 0.158 | 0.383 | | 0.061 | | | 0.162 | 85.42 |  |
| 6 | 0.015 | 0.440 | 0.006 | 0.045 | 0.442 | 0.020 | 0.136 | | 208.73 | 0.053 | 0.468 | | 0.025 | | | 0.109 | 282.36 |  |
| 7 | NA | NA | NA | NA | NA | NA | NA | | NA | 0.026 | 0.486 | | 0.013 | | | NA | NA |  |

Supplement Table 5 Multidimensional Poverty Measures for Persons with and without Dementia by age group with equal nested weights

|  | Age 50-69 | | | | | | | | | | | | | | | |
| --- | --- | --- | --- | --- | --- | --- | --- | --- | --- | --- | --- | --- | --- | --- | --- | --- |
|  | No CID | | | Mild CID | | | | | Moderate/severe CID | | | | | | | |
| d | H | A | M0 | H | A | M0 | P value | % difference in M0 | H | A | | | M0 | P value | | % difference in M0 |
| 1 | 0.838 | 0.186 | 0.156 | 0.946 | 0.210 | 0.198 | <.001 | 27.43 | 1 | 0.236 | | | 0.236 | <.001 | | 51.49 |
| 2 | 0.781 | 0.191 | 0.149 | 0.923 | 0.212 | 0.195 | <.001 | 31.20 | 0.966 | 0.240 | | | 0.231 | <.001 | | 55.32 |
| 3 | 0.255 | 0.281 | 0.072 | 0.462 | 0.273 | 0.126 | <.001 | 76.12 | 0.724 | 0.267 | | | 0.194 | <.001 | | 170.45 |
| 4 | 0.092 | 0.352 | 0.033 | 0.177 | 0.342 | 0.060 | 0.009 | 85.59 | 0.172 | 0.353 | | | 0.061 | 0.159 | | 86.68 |
| 5 | 0.058 | 0.378 | 0.022 | 0.085 | 0.374 | 0.032 | 0.295 | 42.98 | 0.103 | 0.375 | | | 0.039 | 0.337 | | 75.40 |
| 6 | 0.009 | 0.456 | 0.004 | 0.023 | 0.440 | 0.010 | 0.225 | 136.16 | 0.034 | 0.435 | | | 0.015 | 0.229 | | 249.17 |
| 7 | 0.004 | 0.479 | 0.002 | NA | NA | NA | NA | NA | NA | NA | | | NA | NA | | NA |
|  | Age>70 | | | | | | | | | | | | | | | |
|  | No CID | | | Mild CID | | | | | Moderate/severe CID | | | | | | | |
| d | H | A | M0 | H | A | M0 | P value | % difference in M0 | H | | A | M0 | | P value | % difference in M0 | |
| 1 | 0.875 | 0.198 | 0.173 | 1 | 0.229 | 0.229 | 0.002 | 32.33 | 1 | | 0.273 | 0.273 | | <.001 | 57.41 | |
| 2 | 0.813 | 0.204 | 0.166 | 0.878 | 0.246 | 0.216 | 0.017 | 30.38 | 0.957 | | 0.28 | 0.268 | | <.001 | 61.45 | |
| 3 | 0.406 | 0.26 | 0.105 | 0.61 | 0.288 | 0.175 | 0.013 | 66.38 | 0.739 | | 0.318 | 0.235 | | <.001 | 122.72 | |
| 4 | 0.078 | 0.322 | 0.025 | 0.341 | 0.333 | 0.114 | 0.001 | 352.14 | 0.522 | | 0.36 | 0.188 | | <.001 | 646.83 | |
| 5 | 0.031 | 0.338 | 0.011 | 0.146 | 0.379 | 0.055 | 0.023 | 424.96 | 0.348 | | 0.378 | 0.132 | | <.001 | 1146.46 | |
| 6 | NA | NA | NA | 0.049 | 0.449 | 0.022 | 0.076 | NA | 0.087 | | 0.468 | 0.041 | | 0.017 | NA | |
| 7 | NA | NA | NA | NA | NA | NA | NA | NA | 0.043 | | 0.486 | 0.021 | | NA | NA | |
